# Aberrant associations between neuronal resting-state fluctuations and working memory-induced activity in major depressive disorder

**DOI:** 10.1038/s41380-024-02647-w

**Published:** 2024-06-29

**Authors:** Moritz Hempel, Thorsten Barnhofer, Ann-Kathrin Domke, Corinna Hartling, Anna Stippl, Luisa Carstens, Matti Gärtner, Simone Grimm

**Affiliations:** 1 Department of Psychology, MSB Medical School Berlin, Rüdesheimer Straße 50, 14197 Berlin, Germany; 2 School of Psychology, University of Surrey, GU2 7XH Guildford, United Kingdom; 3 Department of Psychiatry and Psychotherapy, Charité – Universitätsmedizin Berlin, Corporate Member of Freie Universität Berlin, Humboldt - Universität zu Berlin, Campus Benjamin Franklin, Hindenburgdamm 30, 12203 Berlin, Germany; 4 Department of Psychiatry, Psychotherapy and Psychosomatics, Psychiatric Hospital, University of Zurich, Lenggstrasse 31, 8032 Zurich, Switzerland

**Keywords:** Neuroscience, Depression, Diagnostic markers

## Abstract

Previous investigations have revealed performance deficits and altered neural processes during working-memory (WM) tasks in major depressive disorder (MDD). While most of these studies used task-based functional magnetic resonance imaging (fMRI), there is an increasing interest in resting-state fMRI to characterize aberrant network dynamics involved in this and other MDD-associated symptoms. It has been proposed that activity during the resting-state represents characteristics of brain-wide functional organization, which could be highly relevant for the efficient execution of cognitive tasks. However, the dynamics linking resting-state properties and task-evoked activity remain poorly understood. Therefore, the present study investigated the association between spontaneous activity as indicated by the amplitude of low frequency fluctuations (ALFF) at rest and activity during an emotional n-back task. 60 patients diagnosed with an acute MDD episode, and 52 healthy controls underwent the fMRI scanning procedure. Within both groups, positive correlations between spontaneous activity at rest and task-activation were found in core regions of the central-executive network (CEN), whereas spontaneous activity correlated negatively with task-deactivation in regions of the default mode network (DMN). Compared to healthy controls, patients showed a decreased rest-task correlation in the left prefrontal cortex (CEN) and an increased negative correlation in the precuneus/posterior cingulate cortex (DMN). Interestingly, no significant group-differences within those regions were found solely at rest or during the task. The results underpin the potential value and importance of resting-state markers for the understanding of dysfunctional network dynamics and neural substrates of cognitive processing.

## Introduction

The symptomatology associated with major depressive disorder (MDD) can be roughly divided into affective, vegetative and cognitive dimensions [1]. The cognitive domain comprises deficits in attention [2], visual learning and memory [3], processing speed [4] and executive functioning [5], such as working-memory (WM) impairments [6]. WM-deficits can be observed even after clinical remission [7, 8] and may have substantial impact on (psychosocial) functioning [9, 10]. Furthermore, WM deficits have been found to negatively predict MDD-treatment outcome [11–13], which underlines the relevance of WM-function as a diagnostic biomarker and potential therapeutic target for individuals with acute or remitted MDD. These findings emphasize the importance of better understanding altered WM-processes in MDD and their underlying neurobiological mechanisms.

Functional magnetic resonance imaging (fMRI) studies of healthy individuals have revealed numerous networks activated during WM, highlighting the crucial involvement of prefrontal and parietal regions, which constitute important nodes of the central executive network (CEN; [14–16]). Furthermore, in healthy controls (HC) significant deactivation of regions within the default mode network (DMN) during WM performance were reported [17, 18]. This is in support of the theory that suppression of these regions, associated with internally-directed and self-referential cognition during periods of task absence [19], is necessary for effective execution of cognitive tasks [20]. A recent meta-analysis of fMRI findings in MDD revealed stronger activation of DMN regions in patients during WM performance [21], which in line with the above reported findings in HC can be interpreted as the failure to adaptively suppress internally-directed cognition for the effective processing of external information [22]. Another meta-analysis of MDD studies [23] reported stronger activation specifically in the left dorsolateral prefrontal cortex (DLPFC) of MDD subjects compared to performance-matched HC, which is in support of the hypothesis that frontal hyperactivation represents a compensatory mechanism to counteract dysfunctional neural activation in other regions to preserve WM performance. Despite these trends, results from studies aimed at delineating differences in WM-related (de-)activation patterns between MDD-patients and healthy control subjects show considerable heterogeneity with various reported regional differences suggesting that our understanding of the neural mechanisms underlying WM deficits in MDD remains incomplete.

In another, hitherto largely unrelated stream of research resting-state fMRI is increasingly employed for the identification of altered neural mechanisms in MDD. Implying involvement of similar regions as in task-based studies, a recent meta-analysis reported large disruptions of resting-state functional connectivity (FC) within and between nodes of the DMN and CEN in MDD patients [24]. Other resting state studies have investigated the amplitude of low frequency fluctuations (ALFF), which quantifies changes of the BOLD signal as a marker of spontaneous neural activity [25]. In MDD increased spontaneous neural activity can be found in the medial prefrontal cortex (mPFC), a core hub of the DMN, and in the insula, which is associated with a coordinative role of switching between the CEN and DMN [26]. Basic research on WM-processes has repeatedly demonstrated associations between WM-performance and functional connectivity between [27, 28] and within these networks [29, 30]. In a machine-learning-based investigation, Xiao et al. [31] revealed, that ALFF values within DMN regions are predictive of visual working memory performance. Furthermore, Zou et al. [32] found that spontaneous neural activity within the superior parietal lobule correlated with performance in a n-back task. These findings suggest a critical role of CEN and DMN regions and their balanced (de-)activation for WM-processing as well as potential alterations of these neurofunctional processes in MDD. Notably, aberrant activity patterns are not only observable during task performance, but network dysfunctionalities may already be present during resting-state periods. In their ‘resting-state hypothesis of depression’, Northoff et al. [33] postulate a comprehensive framework which emphasizes that altered resting-state activity is a key component of MDD, affecting fundamental cognitive and emotional processes that underlie a range of depressive symptoms. This raises the question of how resting-state markers relate to task-evoked activity, and whether any relationship may differ in patients with MDD.

Research in healthy samples has repeatedly demonstrated relations between resting state and task-based assessments. For example, Cole et al. [34] found a strong whole-brain correspondence between the FC during rest- and multiple task-states. Furthermore, there is evidence that task-evoked activation can be predicted by resting state activity using a range of different indices, including fractional ALFF [35], resting-state FC [36, 37] and activity flow over nodes of resting-state networks [38]. Regarding WM-processes more specifically, correlations between spontaneous neural activity and WM-evoked activity of the same region have been reported [32, 39]. These findings suggest the existence of an intrinsic network architecture present during rest [34, 38, 40], which shapes activation patterns following external demands and thereby serves to adaptively prepare the system for effective cognitive processing. Whilst there is a large body of literature addressing either resting-state or task-evoked activity differences in MDD, research investigating MDD-related differences including both modalities or markers of the association between rest- and task-indices is limited [41–47]. Notably, none of these studies calculated parameters of the relationship between resting-state markers and task-evoked activity directly and further conducted group comparisons based on these parameters. The analysis of these neural dynamics in MDD is crucial for a comprehensive understanding how resting-state alterations interact with neural responses to external and cognitive demands, which may shed light on mechanisms underlying cognitive deficits in MDD.

The aim of the current study was to examine the whole-brain relationship between resting state and WM-induced activation in MDD patients and HC using direct parameters. Previous studies revealed distinct patterns of ALFF alterations in MDD [26] as well as a robust relationship between low frequency fluctuations at rest and task-activation in HC [32, 35, 39]. Therefore, the voxel-wise relationship between ALFF at rest and neural activity during an n-back task yields a promising target for the identification of aberrant rest-task markers in MDD and allows for comparison with previous studies in HC. We expected modality-specific group differences and group differences of the rest-task relationship to occur mainly in regions associated with the DMN and CEN.

## Methods

### Participants

The present investigation merged different datasets described in previous publications [22, 48–50] and included 60 subjects currently diagnosed with a depressive episode and 52 HC recruited at the Free University Berlin (FUB). HC were screened for acute or past psychiatric or neurologic conditions. Specific psychiatric exclusion criteria for patients were eating disorders, suicidal ideation, history of substance abuse or dependence and electroconvulsive therapy within the previous 3 months. Concurrent antidepressant medication intake was permitted and recorded.

### Experimental procedure and WM task

All subjects underwent a scanning procedure starting with a T1-weighted anatomical scan, which was followed by a resting-state sequence (8–10 min) and a 2-back task (12 min) with emotional or neutral German nouns as stimuli (EMOBACK-task, [51]). Neutral, positive or negative words were selected from the Berlin Affective Word List [52] and matched based on frequency, imageability, emotional arousal (negative and positive only) and word length. 15 words of the same valence (positive, negative or neutral) were presented block-wise. Each word was displayed for 500 ms with an interstimulus interval of 1500 ms. Participants were instructed to respond by pressing a button on a fiber-optic button box, when the word currently presented was identical to the word 2 trials before. Each block contained 3 target words resulting in 45 correct answers out of 225 words in total. A fixation cross was presented for 10–14 s (fixation condition) after each block. 5 blocks of each valence category were run. The order of the 15 blocks was varied between the subjects by using two parallelized versions of the task. Instructions for the WM task included practice trials outside the MRI. Severity of depression symptomatology was assessed using the Beck Depression Inventory-II [53].

### (f)MRI acquisition

Data was collected on a Siemens Trio 3T. The resting-state sequence at the FUB consisted of 210 or 257 volumes with 37 oblique axial slices of 3 mm (TE = 30 ms; field of view = 192 mm, 3 × 3 mm in-plane resolution, TR 2300 ms, and flip angle 70°). During the WM task the images were collected with 37 oblique axial slices of 3 mm (TE = 30 ms; field of view = 192 mm, 3 × 3 mm in-plane resolution, TR 2000 or 2300 ms, flip angle 70°). A high-resolution anatomical reference image was obtained with a 3-dimensional T1-weighted sequence.

### Preprocessing

The preprocessing of the functional imaging data was performed using standard SPM12 (Wellcome Trust Centre for Neuroimaging, London, UK) routines implemented into the SPM12 toolbox for WM-task data and into the CONN toolbox (conn21a, www.conn-toolbox.org) for the resting state data. Functional images were realigned to the first scan of the session as reference image and motion-related outliers were detected accordingly. The realigned images were coregistered to the mean, which was followed by unified structural and functional segmentation and normalization into standard stereotaxic space (Montreal Neurological Institute 152-brain template). Spatial smoothing was applied using a 6 mm FWHM Gaussian kernel.

### ALFF analysis

For resting-state data, the default denoising procedure implemented into the conn-toolbox was carried out. For each subject a voxel-wise linear regression was performed to remove potential artefacts. Head motion parameters (12 total motion covariates: 6 motion parameters plus 6 temporal derivatives), white matter and cerebrospinal noise factors (5 CompCor eigenvariates for each eroded mask), effects of outlier scans (displacement > 0.9 or global BOLD signal change > 5 SD) and linear BOLD signal trends were included into the model and effects were regressed out. The first 5 scans of the resting-state acquisition were removed, and the following 205 volumes of each participant were included for further analysis, since slightly different lengths (8–10 min) and total numbers of volumes were acquired during rest. The ALFF-value at each voxel was calculated as the root-mean square of the low frequency (0.01–0.1 Hz) power spectrum after applying a temporal band-pass filter [25]. The conn-toolbox was used to create normalized participant-level maps indicating the ALFF value at each voxel in relation to the global ALFF values within that given subject. Two-sample t-tests were conducted for whole-brain group comparison (HC vs. MDD) applying an uncorrected single-voxel threshold of *Z* > 2.3. and a cluster-forming threshold of *p* < 0.05, corrected using gaussian random field (GRF) theory. All cluster-extent based thresholding and GRF correction was carried out using the FSL *cluster* tool (www.fmrib.ox.ac.uk/fsl).

### WM task analysis

For the WM-task data a high pass filter (filter width 128 s) was applied to the time-series to counteract low-frequency drifts. Recent studies of our lab using the Emoback-task had revealed no valence-specific effects in neural activity patterns in MDD patients compared to HC [22]. Additionally, analyses of behavioral data in the current sample did not yield any significant interactions between valence (positive, negative or neutral) and group (Supplementary Table S1). We, therefore, restricted analysis to the contrast between all WM-conditions (regardless of the stimulus valence) versus the fixation condition. Participant-level activation maps of the two contrasts (WM > fixation, fixation > WM) were computed using a fixed-effects voxel-wise GLM in SPM12. The different conditions (WM, fixation) were modeled as box car regressors convolved with a canonical hemodynamic response function. Realignment parameters were included into the model as covariates of no interest. To identify whole-brain group differences (HC vs. MDD), random-effects two-sample t-tests were carried out. The cluster-level threshold was set to *p* < 0.05 (GRF corrected) using an uncorrected single-voxel threshold of *Z* > 2.3.

### Rest-task correlation maps

The analytic approach was partly adapted from previous studies examining the relationship between ALFF and task activity [32, 35, 39]. Within the two groups one-sample t-tests were computed to create masks including regions that were significantly activated or deactivated during the WM condition (single-voxel threshold *Z* > 3.1; cluster significance *p* < 0.05, GRF corrected). The activation and deactivation masks of the MDD group were concatenated with those of the HC. Further analysis of the rest-task correspondence included only regions within those masks to focus regions involved in WM processing exclusively.

The AFNI command *3dTcorrelate* was implemented to calculate the Pearson correlation coefficients between the ALFF and the WM (de-)activation values for each corresponding voxel-pair across the subjects. The maps were computed for each group separately and the abovementioned (de-)activation masks were applied resulting in brain maps indicating the strength of rest-task correspondence at each voxel only within regions significantly activated or deactivated during the WM-task. The corresponding correlation coefficient maps of the MDD patients and HC were Fisher-z transformed to calculate Z-statistics for cluster extent inference. An uncorrected single-voxel threshold of *Z* > 2.3 (cluster significance *p* < 0.05, GRF corrected) was applied to identify clusters showing a significant positive or negative rest-task correlation in either the HC or MDD patients. To compare rest-task correlation coefficients of HC with those of MDD patients, voxel-wise differences of Fisher-z transformed maps (HC – MDD and MDD – HC) were computed to further derive Z-statistics. The corresponding maps were employed for cluster extent inference with a single-voxel threshold of *Z* > 2.3 (cluster significance *p* < 0.05, GRF corrected). More details of the statistical methods and formulae for the rest-task analyses are provided in the Supplementary Methods and Materials.

### Behavioral analysis

We also investigated the relationship between behavioral outcomes and neural activity during either rest or task. Accuracy scores ([hits – false alarms]/targets * 100) and reaction times (RT) were used as indicators of WM-performance. The Pearson correlation coefficients across the subjects between each voxel (of either the ALFF- or the WM-map) and the corresponding RT/accuracy scores were calculated using the AFNI command *3dTcorr1D*. The analysis was carried out for each group separately. An uncorrected single-voxel threshold of *Z* > 2.3 (cluster significance *p* < 0.05, GRF corrected) was employed to identify clusters showing significant brain-behavior correlation.

## Results

### Sample characteristics

No group differences were found regarding sex, age and WM-performance (Table 1). Notably, in the MDD group partial correlation analysis revealed a negative correlation between the BDI and the WM-accuracy whilst controlling for age; *r*(57) = −0.275, *p* = 0.036 indicating weaker performance of individuals with stronger depressive symptoms.

**Table 1:** Demographic and clinical characteristics of the healthy control subjects (HC) and major depressive disorder patients (MDD).

	HC	MDD	Statistics of the group comparisons	*N*
Sex; f/m	23/29	26/34	*X*^2^ = 0.01, *p* = 0.92	*112*
Age; M(SD)	37.6 (10.8)	39.5 (10.6)	*t*(110) = 0.91, *p* = 0.36	*112*
WM Reaction Time (ms); M (SD)	553.2 (182.3)	609.3 (168.7)	*t*(110) = 1.69, *p* = 0.09	*112*
WM Accuracy Score (hits − false alarms/targets * 100); M (SD)	76.0 (27.7)	68.8(20.0)	*t*(110) = 1.59, *p* = 0.11	*112*
BDI	–	31.8 (8.5)	–	111
Antidepressant Medication; yes/no	–	28/30	–	58
Age at Onset of Depression; M (SD)	–	22.8 (10.2)	–	56
Number of Episodes; M (SD)	–	6.13 (5.83)	–	52

*N* represents number of subjects with available information.

### WM-evoked activation and deactivation

Both groups showed similar patterns of WM-elicited activity including significant activation in the bilateral middle and inferior frontal gyrus, bilateral precentral gyrus, bilateral superior and inferior parietal lobule, anterior cingulate cortex, middle temporal gyrus, supplementary motor area, occipital cortex, cerebellum, thalamus, insula, and striatum (Supplementary Fig. S1). Significant deactivation was found in the posterior cingulate cortex, precuneus, medial prefrontal cortex, cuneus, bilateral middle temporal/angular gyrus, bilateral superior temporal gyrus, bilateral Heschl’s gyrus, bilateral postcentral gyrus, calcarine sulcus, bilateral parahippocampal gyrus and bilateral hippocampus (Supplementary Fig. S1).

### Rest-task correlation

**Fig. 1:**
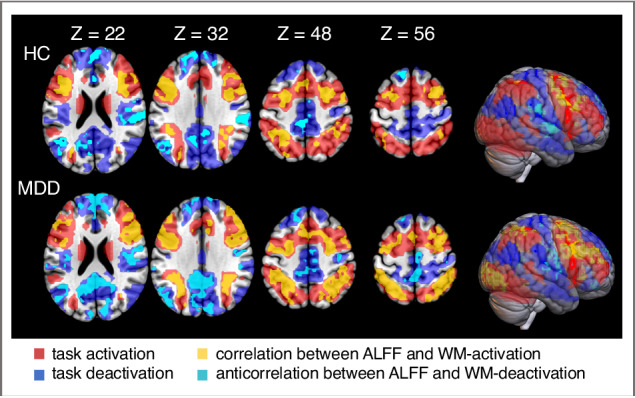
Significant rest-task correlations within both groups. In each group the significant correlations between ALFF during rest and WM-evoked activity are overlaid on the regions where significant activation (red) or deactivation (blue) was captured during the WM task (WM > Fixation, WM < Fixation), single-voxel threshold *Z* > 3.1; cluster significance *p* < 0.05, GRF corrected. Positive correlations between ALFF and WM-activation are shown in yellow, whereas negative correlations between ALFF and WM-deactivation are shown in cyan, single-voxel threshold *Z* > 2.3; cluster significance *p* < 0.05, GRF corrected. The correlation maps were previously masked with the (de-) activation maps, therefore only regions within clusters of significant task-evoked (de-)activation are shown.

In both groups significant rest-task correlations were found in multiple regions, including main hubs associated with the DMN and the CEN (Fig. 1, Table 2). Interestingly, in regions significantly activated by the WM task only positive rest-task correlations occurred (greater ALFF corresponded to greater WM-induced activation), whereas in regions significantly deactivated by the WM task significant negative correlations (greater ALFF corresponded to greater WM-induced deactivation) were found exclusively (Fig. 1, Table 2). Additional analyses confirmed that clusters exhibiting significant rest-task correlation in both groups were mainly located in regions significantly activated or deactivated in either the MDD patients or the HC (Supplementary Fig. S2).

**Table 2:** Cluster characteristics of regions showing significant correlations between ALFF and WM-evoked activity within the HC and MDD patients.

Anatomical region	Number of voxels (27 mm^3^)	Peak *Z*-Statistics	MNI peak coordinates		
			x	y	z
**Healthy controls**					
*Positive correlation*					
MFG/IFG/prCG/SMA/AIC (l & r)	1845	6.85	30	8	55
IPL/SPL (l)	390	6.28	−42	−43	34
IPL/SPL (r)	365	5.61	33	−52	34
MOG/IOG/FG (l)	306	5.22	−39	−61	−5
*Negative correlation*					
MPFC/ACC/SFG (l & r)	866	−5.34	−18	59	28
STG/SMG (r)	452	−6.04	48	−25	28
PCU/PCC/CU (l & r)	433	−5.26	−9	−58	10
AG (l)	171	−5.18	−42	−76	37
**MDD patients**					
*Positive correlation*					
MFG/IFG/prCG/SMA/AIC (r)	1499	6.09	36	2	61
MOG/IOG/FG (l & r)	1296	7.07	−30	−82	−2
MFG/IFG/prCG/SMA/AIC (l)	917	6.69	−36	−1	34
IPL/SPL (l)	840	6.33	−42	−37	34
IPL/SPL (r)	809	7.07	30	−40	37
*Negative correlation*					
PCU/PCC/CU (l & r)	1294	−7.19	−15	−52	16
MPFC/ACC/SFG (l & r)	1015	−6.32	−12	50	−2
STG/SMG/poCG (r)	441	−5.59	48	−22	19
STG/SMG(l)	192	−5.13	−60	−31	31
AG (l)	168	−4.65	−42	−64	25

Cluster-level threshold, *p* < 0.05 (GRF corrected); uncorrected single-voxel threshold of Z > 2.3. min cluster size HC = 80; min cluster size MDD = 108.

*MFG* middle frontal gyrus, *IFG* inferior frontal gyrus, *prCG* precentral gyrus, *SMA* supplementary motor area, *AIC* anterior insular cortex, *IPL* inferior parietal lobule, *SPL* superior parietal lobule, *MOG* middle occipital gyrus, *IOG* inferior occipital gyrus, *FG* fusiform gyrus, *MPFC* medial prefrontal cortex, *ACC* anterior cingulate cortex, *SFG* superior frontal gyrus, *STG* superior temporal gyrus, *SMG* supramarginal gyrus, *PCU* precuneus, *PCC* posterior cingulate cortex, *CU* cuneus, *AG* angular gyrus, *poCG* postcentral gyrus.

### Group differences

Increased WM-elicited activation in MDD was found in two clusters. One cluster was located within the left superior parietal lobule (SPL), inferior parietal lobule (IPL), postcentral gyrus (poCG) and precuneus (PCU), and a second cluster within the right SPL and PCU (Table 3, Supplementary Fig. S3). No other significant group differences emerged regarding WM-evoked (de)activation or ALFF.

**Table 3:** Group differences between HC and MDD patients regarding WM activity, ALFF and ALFF-WM correlation.

Anatomical region of group difference	Direction	Cluster size (voxels)	Peak Z-score	MNI peak coordinates		
				x	y	z
**WM task**						
IPL/SPL/poCG/PCU (l)	MDD > HC	203	3.49	−39	−34	52
SPL/PCU (r)	MDD > HC	187	3.83	15	−49	46
**ALFF-WM correlation**						
DLPFC (l)	HC > MDD	46	4.23	−33	17	25
PCC/PCU (l & r)	MDD > HC	97	4.54	−12	−49	13
MOG/IOG (r)	MDD > HC	106	4.01	30	−88	4

Cluster-level threshold, *p* < 0.05 (GRF corrected); uncorrected single-voxel threshold of Z > 2.3. min cluster size WM Task = 141; min cluster size ALFF-WM correlation = 40.

*IPL* inferior parietal lobule, *SPL* superior parietal lobule, *poCG* postcentral gyrus, *PCU* precuneus, *DLPFC* dorsolateral prefrontal cortex, *PCC* posterior cingulate cortex, *MOG* middle occipital gyrus, *IOG* inferior occipital gyrus.

**Fig. 2:**
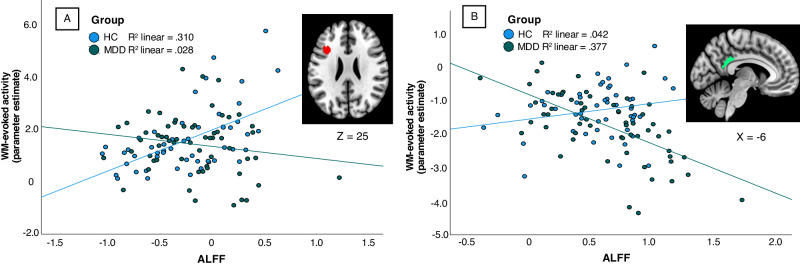
Scatterplots illustrating the group differences of the rest-task relationship. **A** Scatterplot showing increased ALFF-WM correlation in HC in a cluster within the dorsolateral prefrontal cortex. **B** Scatterplot of increased negative rest-task correlation in MDD patients in a cluster within the posterior cingulate cortex/precuneus.

Group differences between rest-task correlation coefficient maps were found within three regions. Compared to the MDD group, stronger positive rest-task correlation in the HC group was identified within a cluster located in the left dorsolateral prefrontal cortex (DLPFC; Table 3, Fig. 2A). MDD patients exhibited greater negative rest-task correlation in a cluster located in the posterior cingulate cortex (PCC)/PCU (Table 3, Fig. 2B). Furthermore, increased positive rest-task correlation of MDD patients was found in a cluster located within the medial occipital (MOG) and inferior occipital gyrus (IOG; Table 3). There were no significant correlations between ALFF and accuracy or RT in the MDD group or the HC group and no significant correlations between WM-evoked activity and accuracy or RT.

## Discussion

The current study revealed a strong relationship between ALFF as an index of spontaneous resting-state activity and activity elicited by a n-back WM-task. While the overall pattern of rest-task relationships was similar across HC and MDD subjects, we found distinct differences in the strength of neural activity and rest-task correspondence between these two groups. MDD subjects revealed stronger WM-evoked activation in bilateral posterior parietal lobules, nodes of the CEN. Furthermore, MDD patients showed a decreased rest-task correlation in the left DLPFC (CEN), an increased negative correlation in the PCC/PCU (DMN) and an increased positive correlation in the MOG/IOG.

### ALFF encodes functional organization properties of the brain

Our study replicates previous findings on rest-task correspondence and, to our knowledge, is the first to extend this research to the investigation of working memory deficits in MDD. Consistent with the results of Zou et al. [32], both HC and MDD participants exhibited a positive relationship between ALFF and task-evoked activation in lateral prefrontal and parietal cortices, core regions for executive functions [54], and a negative rest-task relationship in DMN regions, such as medial-prefrontal, posterior cingulate and lateral parietal regions, where higher ALFF corresponds to stronger deactivation.

This specific pattern of rest-task relationship underpins the theory that resting-state parameters encode important aspects of the functional brain-network organization that guides neural responses to external demands, cognitive processes and behavior [34, 35, 55]. More specifically, ALFF-values may reflect activation potentials elicited in response to specific cognitive demands, which follows the framework of Cole et al. [38] stating that the functional network structure reflects the routes of activity spreading during various cognitive processes. However, spatial [40] and task-dependent specificity [34] of the coherence between resting-state and task-state networks questions if this mechanism translates to complex cognitive or affective processes recruiting subcortical or limbic regions, or if this mechanism is restricted to general aspects of cognitive demanding tasks associated with the DMN and CEN. Further research using other cognitive and affective task-paradigms is needed to validate the generalizability of our findings.

The negative rest-task relationship in DMN regions suggests that subjects with high spontaneous activity at rest exhibit stronger deactivation of these regions during the WM task. Previous research has found activation of the DMN to be associated with rumination [56], self-referential processing [57], internally directed thoughts [58] or mind-wandering [59] and our findings are thus in line with the general observation that DMN suppression is necessary for effective cognitive processing [20].

We found no significant correlations between behavioral outcomes and WM-activity or ALFF, which might be due to the fact that simple linear relationships between behavioral outcomes and activity markers of separated brain clusters are unlikely to account for the complex functional brain organization underlying the successful execution of WM-tasks. Future research may benefit from considering interactional and directional effects between different brain regions during task execution, with special regards to the balanced (de)activation of DMN and CEN regions.

### Neural alterations in MDD

MDD participants showed stronger WM-evoked activation in clusters within the left SPL/IPL/poCG/PCU and within the right SPL/PCU. The posterior parietal cortices, nodes of the CEN, are associated with cognitive control [60, 61] and WM functions [14], such as manipulation of information [62], WM retrieval [63] or storage of verbal information [64]. Previous research with MDD participants has shown increased bilateral [65] as well as decreased right hemispheric activation [66] of parietal regions during WM tasks. In healthy individuals, posterior parietal activation shows associations with WM-load [67, 68] and WM-performance [32, 69, 70]. Similar to the proposed compensatory mechanisms associated with DLPFC hyperactivation in MDD [23, 71, 72], increased parietal activation, as part of the same network, may represent a neural substrate for increased cognitive control exertion or altered coordination of neural and cognitive resources in order to maintain successful WM-performance. In line with this interpretation, previous research has reported increased IPL activation to be associated with cognitive control processes of task-irrelevant emotional stimuli [73]. Furthermore, dynamic casual modeling has revealed inhibitory effects of the right IPL on the MPFC [74]. These findings imply an important coordinative role of the IPL subserving DMN suppression and cognitive control of interfering emotional processes.

MDD patients revealed differences of the rest-task relationship in a cluster located in the PCC/PCU, another major node of the DMN. Besides the involvement during autobiographical memory retrieval [75–77], the PCC is associated with attentional processes and effective behavioral adaption to changing demands of the environment [78–81]. Further, PCC suppression is associated with better performance in various attention-demanding tasks [82–84]. These findings suggest that PCC deactivation supports task-directed attention and awareness and that its activation is a neural reflection of internally directed attention and self-referential mental states which could interfere with task execution [85, 86]. Zou et al. [32] found a stronger negative rest-task relationship in the PCC at higher load conditions. Therefore, our findings may represent increased cognitive demand of the MDD patients, in which increased resting-state activity (increased internally-directed attention) requires stronger suppression during task performance to facilitate compensatory task-directed attention. Recent research revealed that MDD is substantially characterized by increased interrelations between the DMN (including the PCC) and the rest of the brain [87] with task-induced MDD-specific alterations of this relationship [88]. These findings suggest a central involvement of DMN hubs in various cognitive and affective processes and its underlying brain-wide mechanisms specifically altered in MDD [88]. The observed rest-task relationship pattern in the PCC may therefore reflect a neural correlate of global resource allocation to maintain WM-performance in MDD rather than a simple inhibitory regional effect necessary for task execution.

In a cluster within the left DLPFC HC exhibited a stronger rest-task relationship than MDD subjects. If the ALFF at rest encodes activation patterns during task-states, the lack of correlation may be an indication that activation potentials in this region are not elicited properly during WM-performance, potentially disrupting WM processes. We speculate that, since no behavioral deficits were found, compensatory mechanisms in other regions may have counteracted this disruption. Regarding depressive symptomatology, a decreased rest-task relationship may indicate blunted internal representations of external stimuli due to a distorted balance between internally- and externally directed cognition and its corresponding neural processes [33]. Northoff [89] postulates that this imbalance may account for a dysfunctional self-referential focus and ruminative cognition as well as altered exteroceptive processing which may lead to motivational deficits and diminished goal-orientation in depression.

Furthermore, an increased rest-task relationship was found in a cluster within the medial and inferior occipital gyrus. In contrast to the involvement of DMN and CEN regions in MDD, occipital alterations have been reported less frequently. Regarding the involvement of occipital regions in attentional processes [90], we propose that the increased rest-task correlation might be related to heightened demand of attentional focus in MDD.

### Limitations

Some limitations of this study should be mentioned. First, in contrast to many other working memory studies we did not manipulate the cognitive load during the n-back task. Zou et al. [32], for example, observed load-dependent effects of the rest-task relationship in HC. Including different load conditions may contribute to a better understanding of mechanisms underlying the altered rest-task relationship in MDD and reveal more differentiated effects regarding behavioral outcomes. Second, the dichotomous classification of our sample into MDD patients and HC served to provide a strong contrast but ignores the fact that there is considerable heterogeneity within the MDD group. Future studies may want to consider that MDD symptomatology and their neurobiological underpinnings can differ strongly between individuals [91–93]. The fact that we allowed patients with concurrent antidepressant medication into the study may have further increased between-subject variability. It is possible that increased heterogeneity may have contributed to the lack of replication of previous findings, such as the increased frontal activity during WM-performance in MDD subjects [23, 71, 72]. Application of regression models with resting-state parameters and clinical- and demographical covariates as predictors and the task-activation parameters as the dependent variable within a sample of MDD subjects could further advance our presented approach and counteract problems associated with heterogeneity of MDD samples. Adding symptom-based clustering methods of MDD patients might help to reveal symptom-specific alterations of the rest-task relationship. Third, due to our decision to use a whole-brain voxel-wise approach, we were only able to evaluate regional coherence between resting-state fluctuations and task-evoked activity. The question of whether the mechanisms underlying the rest-task relationship (and their alterations) are region-inherent or driven by large-scale network dynamics and top-down processes therefore had to remain unanswered. Since significant rest-task correlations and neural alterations in MDD mainly emerged in regions associated with the CEN or DMN, modulatory effects within or between different networks, as factors causing the rest-task relationship, seem highly likely. For example, future research may want to investigate the coordinative role of the anterior insula in DMN and CEN (de)activation [94] and between-network dynamics by exploring connectivity-based resting-state indices and their relation to task-evoked activation.

## Conclusion

Taken together, these findings suggest that resting-state activity reflects important properties of WM processes and their neural representations. The fact that a consistent pattern of correlations was found across HC and MDD-patients underlines the applicability and relevance of resting-state data for the understanding of brain functionality. Most importantly, analysis of rest-task relationships identified meaningful MDD-associated differences involving main hubs of the CEN and DMN that would have remained unnoticed in analyses of separate parameters. In conclusion, the integration of rest- and task data with parameters of their relationship offers an avenue to gain a more comprehensive understanding of the processes underlying cognitive deficits and network mechanisms altered in MDD.

## Supplementary information

Supplemental Material

## Data Availability

All data analyzed during the current study are available from the corresponding author upon reasonable request.
