# Supplementary material for: Aberrant associations between neuronal resting-state fluctuations and working memory-induced activity in major depressive disorder
Source: Mol Psychiatry. 2024 Jun 29;30(1):4–12. doi: 10.1038/s41380-024-02647-w (PMC11649556; doi:10.1038/s41380-024-02647-w)
Supplement: Supplementary file 1 — Supplemental Material [file 41380_2024_2647_MOESM1_ESM.docx]

**Supplementary Methods and Materials**

**Rest-task analyses**

To derive voxel-wise Z-statistics for each group (MDD and HC) indicating the strength and statistical significance of the Pearson correlation coefficients (*r*) between the ALFF maps and WM-(de)activation maps, the following formula was applied:

$$Z= \frac{\frac{1}{2} ln \left( \frac{1+r}{1-r} \right)}{\sqrt{\frac{1}{n-3}}}$$

Voxel-wise Z-statistics of group differences between HC and MDD patients were computed with the following formula, where *z_x_* and *z_y_* represent Fisher-z transformed correlation coefficients of either MDD patients or HC:

$$z_{x}= \frac{1}{2} ln \left( \frac{1+r_{x}}{1-r_{x}} \right)$$

$$z_{y}= \frac{1}{2} ln \left( \frac{1+r_{y}}{1-r_{y}} \right)$$

$$Z= \frac{z_{x}- z_{y}}{\sqrt{\frac{1}{n_{x}-3}+ \frac{1}{n_{y}-3}}}$$

**Supplementary Figures**

**Figure S1** Regions showing significant activation (red) or deactivation (blue) during the WM condition (WM>Fixation, WM<Fixation) in either the HC or MDD group, single-voxel threshold *Z* > 3.1; cluster significance *p* < .05, GRF corrected.

**Figure S2** This figure illustrates the rest-task relationship in regions where significant (de)activation was elicited during the WM-task (orange for positive and green for negative correlations) in contrast to correlations in regions where significant task-evoked (de-)activation was not found, yellow for positive and cyan for negative.

**Figure S3** Regions showing significant group differences of WM-activation, single-voxel threshold *Z* > 2.3; cluster significance *p* < .05, GRF corrected.

**Supplementary Tables**

**Table S1**: Results of the ANOVAs for the reaction time (RT) and accuracy scores of the N-back task with the factors Group (HC/MDD) and valence (positive/negative/neutral) of the presented words.

| **Effect** | ***df*** | ***F*** | ***p*** |
| --- | --- | --- | --- |
| **RT** |  |  |  |
| Group | 1, 110 | 2.87 | .09 |
| Valence | 2, 220 | 0.35 | .71 |
| Group x Valence | 2, 220 | 2.47 | .09 |
| **Accuracy** |  |  |  |
| Group | 1, 110 | 2.51 | .12 |
| Valence | 2, 220 | 0.79 | .46 |
| Group x Valence | 2, 220 | 1.29 | .28 |
